# Effects of ECM proteins (laminin, fibronectin, and type IV collagen) on the biological behavior of Schwann cells and their roles in the process of remyelination after peripheral nerve injury

**DOI:** 10.3389/fbioe.2023.1133718

**Published:** 2023-03-24

**Authors:** Peng Yu, Guanhua Zhang, Bo Hou, Enpeng Song, Jiaming Wen, Yueyang Ba, Donglin Zhu, Gangwei Wang, Feng Qin

**Affiliations:** ^1^ Department of Neurosurgery, The Third Affiliated Hospital, Sun Yat-sen University, Guangzhou, Guangdong, China; ^2^ Department of Cerebrovascular Surgery, The Third Affiliated Hospital, Sun Yat-sen University, Guangzhou, China; ^3^ Department of Obstetrics, Guangzhou Women and Children’s Medical Center, Guangzhou Medical University, Guangzhou, Guangdong, China; ^4^ Department of Clinical Laboratory, The Third Affiliated Hospital of Sun Yat-sen University, Guangzhou, China; ^5^ Department of Emergency, The Third Affiliated Hospital of Sun Yat-Sen University, Guangzhou, Guangdong, China

**Keywords:** ECM proteins, Schwann cells, peripheral nerve injury, myelination, regeneration

## Abstract

**Introduction:** It is important to note that complete myelination and formation of myelinated fibers are essential for functional nerve regeneration after peripheral nerve injury (PNI). However, suboptimal myelin regeneration is common and can hinder ideal nerve regeneration. Therefore, it is important to closely monitor and support myelin regeneration in patients with PNI to achieve optimal outcomes.

**Methods:** This study analyzed the effects of three extracellular matrix (ECM) proteins on Schwann cells (SCs) in the nerve regeneration environment, including their adhesion, proliferation, and migration. The study also explored the use of composite sodium alginate hydrogel neural scaffolds with ECM components and investigated the effects of ECM proteins on remyelination following peripheral nerve injury.

**Results:** The results showed that laminin (LN), fibronectin (FN), and collagen Ⅳ (type IV Col) promoted the early adhesion of SCs in 2-dimensional culture but the ratios of early cell adhesion were quite different and the maintenance of cells’ morphology by different ECM proteins were significantly different. In transwell experiment, the ability of LN and FN to induce the migration of SCs was obviously higher than that of type IV Col. An vitro co-culture model of SCs and dorsal root ganglia (DRG) neurons showed that LN promoted the transition of SCs to a myelinated state and the maturation of the myelin sheath, and increased the thickness of neurofilaments. Animal experiments showed that LN had superior effects in promoting myelin sheath formation, axon repair, and reaching an ideal G-ratio after injury compared to FN and Col IV. The situation of gastrocnemius atrophy was significantly better in the LN group. Notably, the thickness of the regenerated myelin sheaths in the type IV Col group was the thickest.

**Conclusion:** In this experiment, we analyzed and compared the effects of LN, FN, and type IV Col on the biological behavior of SCs and their effects on remyelination after PNI and further clarified their unique roles in the process of remyelination. Further research is necessary to explore the underlying mechanisms.

## 1 Introduction

After peripheral nerve injury (PNI), remyelination is a critical step for functional nerve regeneration (Chooi and Chew, 2019). Functional nerve regeneration relies on the reinnervation of target tissues by regenerating axons, which is an essential process. In addition, regenerated axons can only function properly after undergoing myelination and forming myelinated fibers. The presence of myelin tissue is necessary for the rapid and effective conduction of nerve signals (Salzer, 2015; Belin et al., 2017). However, unsatisfactory remyelination is a common problem after PNI, hindering the achievement of ideal nerve regeneration. Currently, there is no specific and effective method to promote remyelination after PNI. Thus, investigating the internal factors that affect remyelination has been a focus in this field.

The extracellular matrix (ECM) is a complex network of molecules that provides structural support for cells and regulates intercellular communication (Xu et al., 2020). Through its 3-dimensional structure, the ECM aligns cells and creates physical pathways for cell movement. Additionally, it can interact with various molecules, such as growth factors, signal receptors, and adhesion molecules, to produce a range of effects on cellular behavior and function. ECM macromolecules are intercellularly deposited in the peripheral nerve microenvironment, providing a suitable niche for the growth of Schwann cells (SCs). Myelin, a multilayered extension of the glial membrane surrounding axons, is crucial for proper nerve function. Schwann cells play a crucial role in the regeneration of the myelin sheath, with their proliferation, migration, dedifferentiation, phenotype, and gene expression all directly impacting this process. Additionally, Schwann cells are responsible for forming the “bands of Büngner” which act as guides for growth cones through the distal stump of a damaged nerve (Namgung, 2014); SCs play a crucial role in nerve regeneration, and their interaction with ECM molecules is essential for proper myelin formation around axons, as evidenced by several studies (Ard et al., 1987; Ning et al., 2019). Understanding the impact of ECM could provide valuable insights for improving the process of remyelination.

The ECM of peripheral nerves typically contains collagen Ⅳ, laminin, and fibronectin, which are non-collagen glycoproteins (Su et al., 2021). The ECM and SCs are intimately associated with the nerve regeneration environment under both physiological and pathological conditions; for instance, laminin regulates Schwann cell proliferation and survival (Chen et al., 2005) and is an important factor in promoting the radial sorting of SCs (Chernousov et al., 2008) and the formation of myelin. Collagen and its receptors can promote the adhesion, migration, myelination of SCs and neurite growth (Klein et al., 2016; Mehta and Piao, 2017). Fibronectin can promote SC growth and motility (Mosahebi et al., 2003). Tissue engineering has been employed to design composite scaffolds with diverse ECM components to facilitate the repair of damaged peripheral nerve tissue, yielding promising outcomes. Notably, these designs prioritize the impact on axons (Novikova et al., 2006; Armstrong et al., 2007; Gonzalez-Perez et al., 2013). However, the distinctive contributions of ECM components to SCs remain elusive, and their effects on the remyelination process require further elucidation.

This study investigated the influence of three ECM components, laminin, fibronectin, and type IV collagen, on the biological behavior of SCs and the effect of their composite hydrogels (sodium alginate) on myelin regeneration after PNI. And also explored the unique roles of these ECM components in the remyelination process.

## 2 Materials and methods

### 2.1 Animals

8-week-old male Sprague Dawley rats (body weight 150–200 g) were housed in an animal facility for at least 7 days prior to surgery. All experimental animals (provided by the Experimental Animal Center of the School of Medicine, Sun Yat-sen University) were housed in temperature- and humidity-controlled animal quarters with a 12-h light/dark cycle. All protocols were approved by the Experimental Animal Administration and Ethics Committee of Sun Yat-sen University, Guangdong, China.

### 2.2 Cell culture

S16 cell line was purchased from ATCC (Manassas, VA) and maintained with Dulbecco’s modified Eagle’s medium (DMEM) containing 10% FBS (Gibco, Grand Island, NY) supplemented with 100 IU/mL penicillin G and 100 mg/mL streptomycin at 37°C in a humidified 5% CO_2_ atmosphere. Before conducting the Schwann cell-related experiments, we identified the S16 cell line (Supplementary Figure S1).

First, an 8-week-old adult SD rat is selected, and the skin is removed from the back to expose the spine (Ning et al., 2020). These ganglia are treated with collagenase and trypsin and subjected to density gradient centrifugation (5 min, 200 g) using 30% Percoll PLUSTM to remove non-neuron cells; Subsequently, the extracted DRG neurons are seeded at a density of 1.5 × 10^3^ cells per well in a 6-well plate that has been precoated with ECM protein on a 12 mm × 12 mm square coverslip.

DRGs were cultured in neurobasal medium containing B27 (GIBCO, Carlsbad, CA), 0.08% glucose, 50 ng/mL NGF (Harlan Bioproducts, Indianapolis, Indiana) and 15 µM 5-fluorodeoxyuridine (FdUr) (Sigma-Aldrich, St. Louis, MO).

When the dendrites of DRG neurons extend to the border of the cover glass, SCs were seeded on neurons at a density of 1.5 × 10^5^ cells/slide and were cultured in minimum essential medium (MEM) (Mediatech, Manassas, VA) supplemented with 10% fetal bovine serum (Mediatech, Manassas, VA), 0.08% glucose and 50 ng/mL NGF. After 7 days, the medium was replaced with myelin culture medium. MEM was supplemented with 10% fetal bovine serum, 0.08% glucose, NGF (50 ng/mL) and ascorbic acid (50 µg)/ml. Continue to cultivate for 7 days, immunofluorescence staining was used to evaluate myelination (MBP) and neurofilament (NF-200) growth.

### 2.3 Detection of SC biological behavior

#### 2.3.1 Cell proliferation assay

SCs were seeded on glass coverslips precoated with laminin (2 μg/mL), fibronectin (2 μg/mL), collagen IV (5 μg/mL) (all from Sigma) and PBS (1x) at a seeding density of 1 × 10^4^/slide and routinely cultured for 48 h. We first cultured DRG neurons and SCs separately with different concentrations of LN coated in low-adhesion culture dishes. The lowest coating concentration where both cell types could adhere and have good survival (Live/dead assessment was performed to evaluate) was used. SC proliferation was assessed using the 5-ethynyl-2′-deoxyuridine (EdU) proliferation assay. EdU was added to the cultures at a final concentration of 20 μM following the manufacturer’s instructions and incubated at 37°C for 24 h. The cells were fixed with 4% paraformaldehyde in PBS for 30 min, and after fixed, SCs were analyzed using a Cell-Light EdU DNA Cell Proliferation Kit (RiboBio) following the manufacturer’s protocol. A DMR fluorescence microscope (Leica Microsystems, Bensheim, Germany) was used to calculate the number of EdU-positive Schwann cells/total number of SCs in images of randomly selected fields of view. Assays were performed 3 times using triplicate fields of view.

#### 2.3.2 Transwell migration experiment

SCs migration assays were performed using a BD BioCoat cell culture insert with a 6.5 mm diameter filter and 8 µm pore size following the manufacturer’s instructions (BD Biosciences). First, laminin (2 μg/mL), fibronectin (2 μg/mL), type IV collagen (5 μg/mL) (all from Sigma), and PBS (1x) were preplated on polycarbonate membranes (membrane between the upper and lower chambers to which SCs could attach) and then SCs were seeded (1.5 × 10^5^ cells/insert) into the upper chamber containing complete medium supplemented with 2 mM L-glutamine, 50 U/mL penicillin, 50 mg/L streptomycin and 1% DMEM high glucose medium (v/v). Complete medium containing UTP was placed in the lower chamber as a chemoattractant. After 24 h of incubation in a humidified environment with 5% CO_2_ at 37°C, cells that migrated to the lower chamber were fixed with 70% (v/v) ethanol and stained with 0.2% (w/v) crystal violet. For each polycarbonate membrane, the number of cells was counted under a light microscope in 4 randomly selected random fields. The number of migrated cells in the Control group (PBS) was used as a reference value of 1.

### 2.4 Evaluation of the SC-DRG coculture model

#### 2.4.1 Enzyme-linked immunosorbent assay (ELISA) to measure the levels of myelin-associated protein (MAG) and nerve cell adhesion molecule 1 (NCAM-1)

Levels of MAG and NCAM-1 were analyzed using a MAG and NCAM-1 ELISA kit (BD Biosciences, San Jose, CA, United States) following the manufacturer’s instructions. Through preliminary experiments, it was determined that 4 × 10^5^ cells would be used for each detection. After cell lysis, protein extraction was performed and 100 µL of the extract was added to each well of a 96-well plate. The plate was incubated with the primary antibody (100 µL) at 37°C for 1 h, followed by thorough washing and incubation with the secondary antibody (100 µL) at 37°C for 30 min in the dark. After incubation, 100 µL of TMB substrate solution was added to each well and the plate was incubated at 37°C in the dark for 20 min for color development. Then, 100 µL of stop solution was added to each well, which changed the color from blue to yellow. Within 10 min, the absorbance (OD value) of each well was measured at 450 nm using an ELISA reader.

#### 2.4.2 Immunofluorescence staining

DRG-SCs were washed in PBS and then fixed with 4% paraformaldehyde for 20 min. After washing by PBS, the samples were permeabilized in ice-cold methanol for 25 min and then blocked with 5% normal goat serum + 0.3% Triton X-100 at room temperature for 1 h. Then, the cells were incubated overnight with myelin basic protein (MBP) (mouse-derived) primary antibody prepared in blocking solution. After washing with PBS, the cells were incubated with NF-200 (rabbit-derived) primary antibody overnight. The cells were incubated with Alexa 594-conjugated goat anti-mouse secondary antibody (Invitrogen) and Alexa 488-labeled goat anti-rabbit secondary antibody (Invitrogen) for 45 min each. Nuclei were stained with DAPI. After mounting with antifade medium, myelin fragments were observed by epifluorescence on a Nikon E800 microscope.

#### 2.4.3 Western blot was performed to detect myelin-related transcription factors in the co-culture system (Krox 20, Sox 10 and Oct 6)

The cells at co-culture system were lysed in RIPA buffer containing 1% SDS and protease inhibitor (aprotinin, leupeptin, pepstatin A, PMSF). The protein concentration of cell lysates was measured by using Bradford assay. The samples were prepared in an SDS sample buffer, heated for 3min at 98°C, and 6 μg of the protein from each sample were loaded in SDS loading buffer and then transferred to a PVDF membrane. The membranes were blocked in 2.5% skim milk for 1 h, followed by incubation with anti-rabbit Krox20 (1:1000, Abcam),anti-rat Sox 10 (1:800, Abcam) or anti-rat MBP (1:500, Abcam) antibodies at 4°C overnight. The membranes were washed three times in TBST and incubated with goat anti-rabbit and anti-rat IgG conjugated to horseradish peroxidase (1:1000, Sigma) for 2 h. Bands were visualized by using ECL system.

### 2.5 Cultivation of SCs under 3-dimensional conditions

Sodium alginate (W201502-SAMpLE, Sigma) was mixed with FN and then mixed with growth medium (1:1) to obtain alginate-ECM hydrogel with a final concentration of 2% alginate and 0.05% FN. Then, 48-well plates were precoated with 100 µL of mixed solution. The growth medium was replaced with medium containing 1 × 10^7^/mL SCs to obtain an alginate-ECM-SCs hydrogel, and 100 µL was added to the wells precoated with hydrogel. After the gel formed, 200 µL of growth medium was added, and 100 µL of medium was aspirated every day and replaced with equivalent fresh medium. Alginate-ECM hydrogels of LN (10 μg/mL, 114956-81-9, Sigma) and type IV collagen (2 mg/mL, 11179179001, Sigma) were obtained using the same method. Sodium alginate hydrogel was found that almost complete degradation occurred after 4 weeks. The degradation curve is shown in Supplementary Figure S2.

#### 2.5.1 Live/dead staining

Cell viability was assessed using a live/dead cell staining kit (Beyotime, China). SCs were mixed with the above sodium alginate hydrogels at a density of 1 × 10^7^ cells/mL and seeded into confocal culture dishes. After 72 h of culture, Calcein AM/pI detection working solution was prepared following the manufacturer’s instructions and added to the culture system. Imaging was performed using a Leica TCS Sp8 X white light laser confocal microscope (Leica Microsystems GmbH, Wetzlar, Germany).

### 2.6 Surgical procedure for the animal model

**FIGURE 1:**
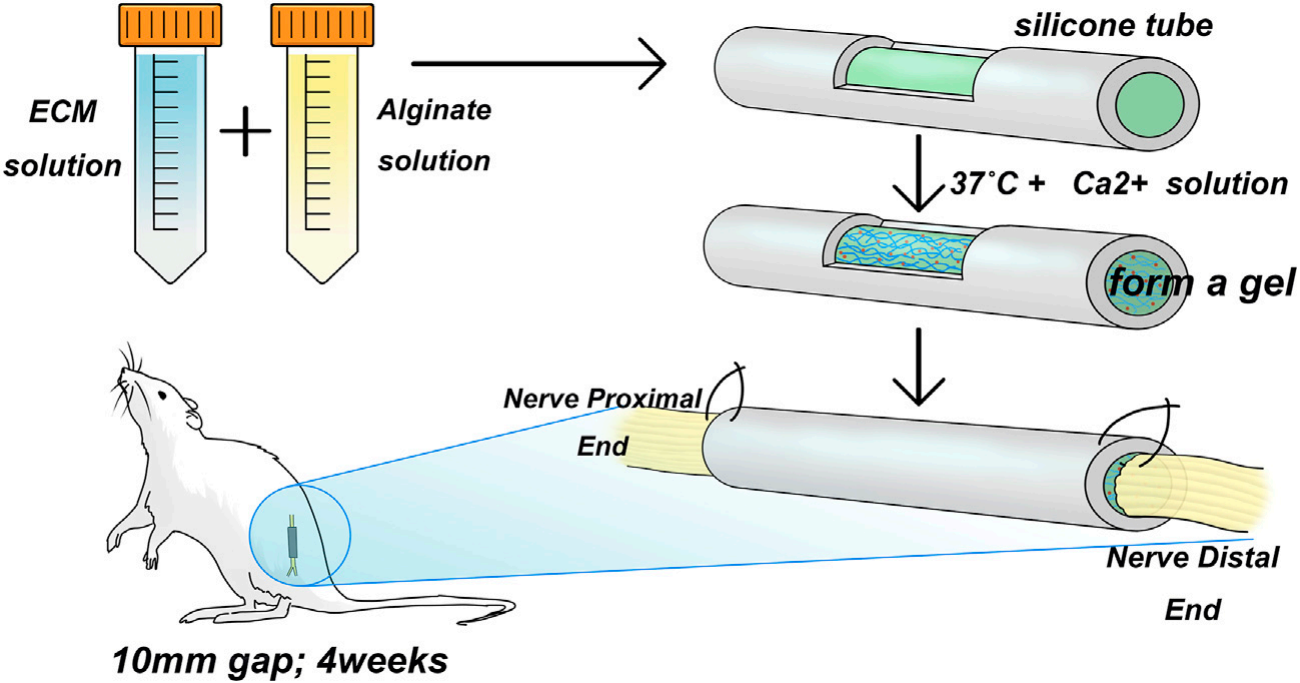
Animal modeling diagram. The ECM solution and alginate hydrogel were mixed and introduced into a silicone tube, followed by the addition of a Ca^2+^ salt solution at 37°C to form a gel. The tube was then used to bridge a 1 mm sciatic nerve gap in adult SD rats by suturing.

Twenty SD rats were randomly assigned to Sham group, LN group, FN group, type IV Col group and Control group. Each rat was injected with a saline solution containing 15 mg/g atropine (per gram animal body weight, Pfizer Pharmaceuticals Ltd., New South Wales, Australia) and 1 mg of xylazine hydrochloride (Troy Laboratories Ltd., NSW, Australia) and then anesthetized with 3% isoflurane (Baxter, NSW, Australia) in oxygen at 1.0 L/min. The skin was incised, the muscle was exposed, and 1 side of the sciatic nerve was isolated. At a distance of 0.5 cm from the tibial and common peroneal nerve branch, the sciatic nerve trunk was cut, and a 1-cm segment of the sciatic nerve was cut in the proximal direction. The silicone tubes for the LN, FN, and type IV Col groups were filled with LN, FN, and type IV Col sodium alginate complexes, respectively, and silicone tubes for the group without ECM hydrogel were filled with only sodium alginate hydrogel. Simply put, the ECM molecule-loaded sodium alginate hydrogel is loaded into a silicone tube, and the ends of the silicone tube are sutured to the proximal and distal ends of the severed sciatic nerve (Figure 1). All animals were housed in a standard environment. All surgical procedures were designed to reduce the number of rats and their suffering. Experimental animals were euthanized using inhaled ether.

### 2.7 Postoperative assessment of remyelination and neurological recovery

#### 2.7.1 Transmission electron microscopy (TEM) and toluidine blue (TB) staining

Four weeks after surgery, the grafts were removed from the rats, and the nerves were isolated by removing the surrounding adipose tissue and blood vessels. The specimens were fixed with 2.5% glutaraldehyde at 4°C for 6 h and washed with 0.1 mL/L of PBS (pH 7.2) six times for 3 min per wash. Then, the specimens were post-fixed in 1% osmium tetroxide at 4°C for 2h, dehydrated in a graded series of acetone, embedded with Poly/Bed 812 resin (Ted Pella, CA, United States) and polymerized at 60°C for 48 h.Ultrathin sections (60–80 nm) prepared with an ultramicrotome (Leica EM UC7-Leica) were mounted onto a 200-mesh formvar-coated copper grid, stained with uranyl acetate and lead citrate and examined under a TEM (FEI Tecnai Spirit G 2). Twelve ultrathin sections (6 for TEM and 6 for TB) were prepared for each group.

Analysis of TEM and TB staining data: Image-Pro Plus (IPP) software was used to analyze axon diameter, number of myelinated fibers, thickness of the myelin sheath and G-ratio. The G-ratio is the ratio of the inner diameter to the outer diameter of the myelin sheath of myelinated axons, serving as a good parameter for evaluating the thickness of the myelin sheath in the process of peripheral nerve regeneration (Fraher, 1992).

$$G-ratio=AD/\left(AD⊕MT\right)$$

AD: axon diameter; MT: myelin sheath thickness.

The above procedure was performed by a skilled pathologist without knowledge of the experimental design.

#### 2.7.2 Immunofluorescence staining of graft specimens

After obtaining fresh specimens, the specimens were fixed in 4% paraformaldehyde for 12 h, with a fixed liquid to specimen ratio of 10:1. 5-10-µm-thick tissue cryosections of the middle part of the grafts were prepared. Sections were incubated with anti-neurofilament 200 (NF-200; Sigma; 1:2000, St. Louis, MO, United States) and anti-MBP(Abcam; 1:800, Cambridge, MA, United States), Alex Fluor 488-conjugated (for NF-200) or Alex Fluor 594-conjugated (for MBP) secondary antibodies were used for detection. Images were acquired with a fluorescence microscope (Eclipse 80i, Nikon) equipped with a high-resolution color digital camera (Digital Sight US-U2, Nikon). The results were analyzed using the IPP software (Media Cybernetic, Inc.).

#### 2.7.3 ELISA was used to measure the expression of negative regulators of myelin formation, EphA4 and Ninj2, in the transplanted specimens *in vivo*

Once the transplant samples were obtained, they were homogenized in an equal amount of physiological saline. The homogenates were centrifuged at 1,000 rpm for 10 min and the supernatants were collected. The OD values (450 nm) were determined using the EPHA4 ELISA kit (LM8H0831, LMAIBio.China) and Ninjurin-2 ELISA kit (E13393r, EIAAB SCIENCE INC. WUHAN), according to the manufacturer’s instructions.

#### 2.7.4 Muscle wet weight and histological analysis

The gastrocnemius muscle innervated by the sciatic nerve was weighed to determine postoperative atrophy of the innervated muscle. After the rats were sacrificed by deep anesthesia, gastrocnemius muscles of the left and right limbs were collected and quickly weighed on an accurate scale. The relative gastrocnemius muscle weight (ratio of the muscle weight of the experimental side to the muscle weight of the control side) was calculated. After weighing, the gastrocnemius muscle was immersed in 4% polyaldehyde overnight, washed, dehydrated with a graded ethanol series, and embedded in paraffin. The muscle was cut into 5-μm thick sections for Masson staining. Images were acquired with a fluorescence microscope (Eclipse 80i, Nikon) equipped with a high-resolution color digital camera (Digital Sight US-U2, Nikon). The cross-sectional area of muscle fibers was calculated using IPP software (Media Cybernetic, Inc.).

#### 2.7.5 Dynamically monitoring the sciatic functional index (SFI) after surgery

Randomly selecting 3 rats per group, SFI was measured and recorded at 0, 1, 2, 3, and 4 weeks after surgery: Measure the experimental paw length (EPL), intermediate toe spread (EIT) and toe spread (ETS) of the exprimental limb. Measure the normal paw length (NPL), intermediate toe spread (NIT) and toe spread (NTS) of the contralateral limb. Measure the normal paw length (NPL) and toe spread (NTS) of the contralateral limb calculate the SFI using the formula: SFI = −38.3*(EPL − NPL)/NPL + 109.5*(ETS − NTS)/NTS + 13.3*(EIT − NIT)/NIT − 8.8.

### 2.8 Statistics

All results are expressed as the mean ± standard deviation. To analyze intergroup differences, one-way analysis of variance (ANOVA) followed by Tukey’s multiple comparison test (*α* = 0.05) was performed in the statistical software GraphPad Prism 9.0.

## 3 Results

### 3.1 Effects of LN, FN, and type IV Col on SCs’ adhesion and proliferation under 2D culture

#### 3.1.1 SCs adhesion

**FIGURE 2:**
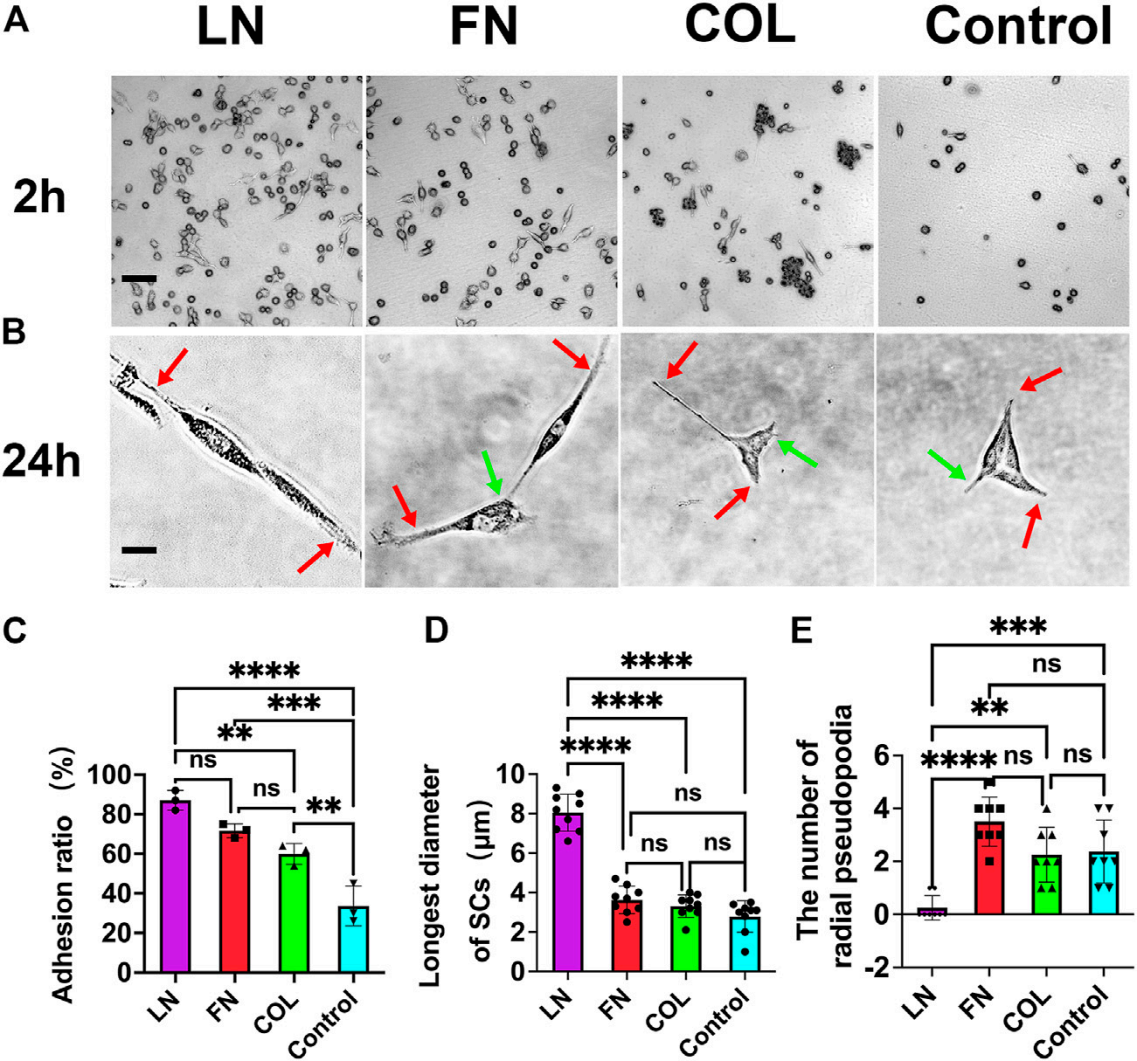
Adhesion and cell morphology of SCs in early 2D culture conditions. The adhesion of SCs at 2 h after seeding **(A)** and the morphology of SCs at 24 h after seeding **(B)**: axial pseudopodia (red arrow) and radial pseudopodia (green arrow). The adhesion ratio in the LN group was significantly higher than that in the other groups (*p* < 0.01) [**(C)**, *n* = 3]. Major axis of SCs (the maximum length of a single cell) in the LN group was significantly larger than that in the FN group and type IV Col group (*p* < 0.0001) [**(D)**, *n* = 9]. The number of radial pseudopodia of SCs in the LN group was significantly less than that of SCs in the FN group and IV Col group (*p* < 0.01) [**(E)**, *n* = 8]. **p < 0.01, ***p < 0.001, ****p < 0.0001, ^ns^ *p* > 0.05. One-way ANOVA with Tukey’s *post hoc* test. Scale bar **(A)** = 10 µm. Scale bar **(B)** = 1 µm.

SCs were cultured on 12 mm × 12 mm glass coverslips coated with laminin (LN), fibronectin (FN), or type IV collagen (type IV Col), as well as on blank glass coverslips for 2 h (Figure 2A). The cell adhesion ratio for LN-coated coverslips (87% ± 5%) was slightly higher than that for FN-coated coverslips (72% ± 4%) (*p* = 0.0747) and significantly higher than that for type IV Col-coated coverslips (60% ± 5%) (*p* = 0.0039). The cell adhesion ratio for the LN, FN, and type IV Col groups was significantly higher than that for the blank group (34% ± 10%) (*p* < 0.01), indicating that these ECM components can enhance the early adhesion of SCs (Figure 2C).

All four groups exhibited 100% cell adhesion 24 h after seeding, but the cells in the FN and type IV Col groups had a polygonal shape, while the cells in the LN group were more slender and spindle-shaped (Figure 2B). The major axis in the LN group was significantly higher than the other three groups, as determined by analysis using IPP software (Figure 2D). Specifically, the maximum cell diameter in the LN group was 8.0 ± 0.9 μm, whereas the maximum cell diameters in the FN, type IV Col, and Control groups were 3.6 ± 0.7, 3.3 ± 0.6, and 2.8 ± 0.8 μm, respectively (*p* < 0.0001).

The number of axial pseudopodia on the SCs in the 4 groups was 2, as shown by red arrows in Figure 2B (red arrow). The number of radial pseudopodia of SCs in the 4 groups was found to differ significantly (Figure 2E). Specifically, SCs in the LN group had significantly fewer radial pseudopodia (0.3 ± 0.5) compared to the FN group (3.5 ± 0.9, *p* < 0.0001), the type IV Col group (2.3 ± 1.0, *p* = 0.0012), and the Control group (2.4 ± 1.2, *p* = 0.0006). The number of radial pseudopodia, which are cell protrusions other than pseudopodia from the axial direction (green arrow in Figure 2B), was not significantly different among the FN, type IV Col, and Control groups (*p* > 0.05).

#### 3.1.2 SC proliferation

**FIGURE 3:**
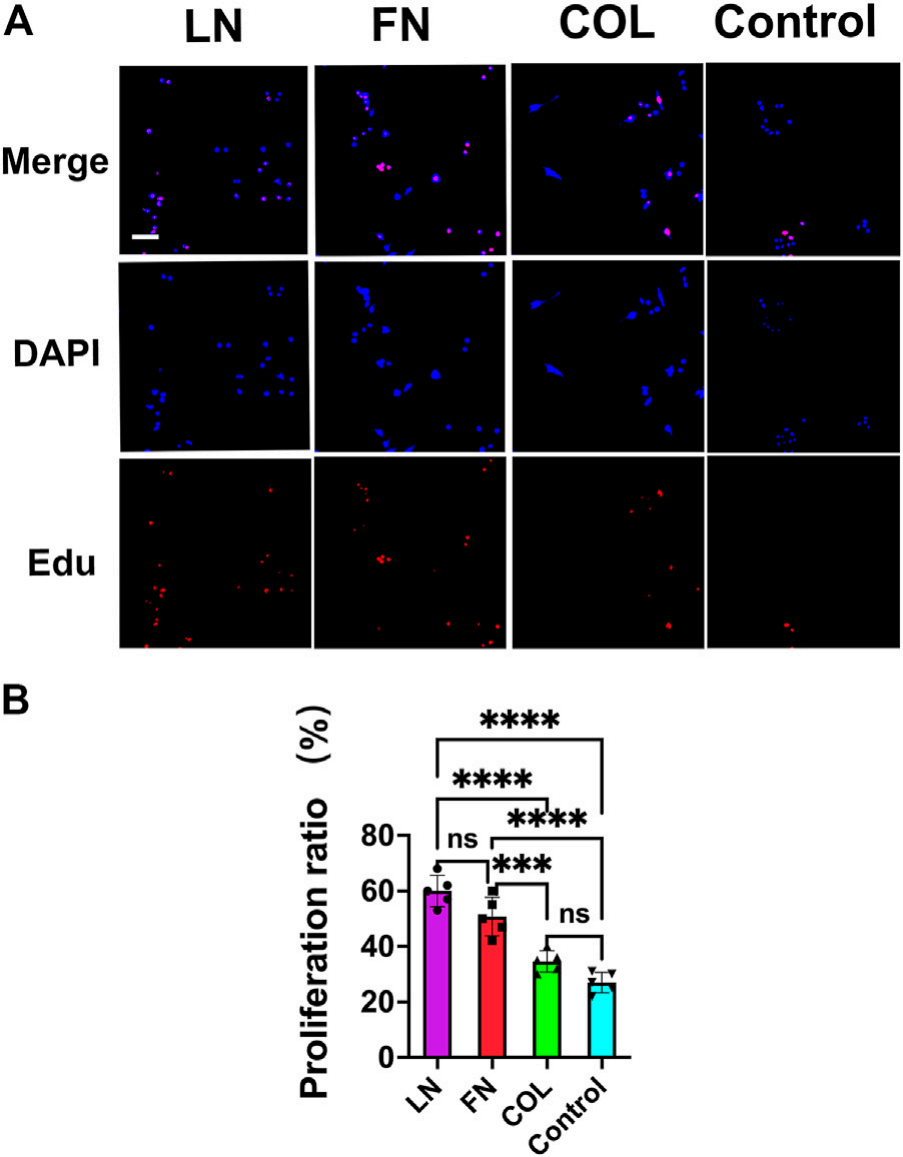
The proliferation of SCs under 2D culture conditions (EdU staining). Twenty-four hours after seeding SCs, the proliferation of SCs in the LN, FN and type IV Col groups were observed under a fluorescence microscope**(A)**, and the proportion of proliferating cells was quantified [**(B)**, *n* = 5]. The proportion of proliferating cells (red) in the LN group and FN group was significantly greater than that in the type IV Col group and Control group (*p* < 0.0001). ***p < 0.001, ****p < 0.0001, ^ns^ *p* > 0.05. One-way ANOVA with Tukey’s *post-hoc* test. Scale bar = 4 µm.

SCs were seeded on glass coverslips (12 mm*12 mm) precoated with LN (2 μg/mL), FN (2 μg/mL), type IV Col (5 μg/mL) (all from Sigma) or PBS (Control group) at a seeding density of 1 × 10^4^/slide. EdU staining was performed after 24 h of conventional culture (Figure 3A red), and DAPI was used to counterstain cell nuclei (Figure 3A blue). The proportion of SCs in the proliferative state (red) was quantitatively analyzed (Figure 3B). In the LN group, 60% ± 6% of SCs were in the proliferative state, slightly higher than that in the FN group (51% ± 7%) (*p* = 0.0569). In both the LN and the FN groups, the proportion of SCs in the proliferative state was significantly higher than that in the type IV Col group (35% ± 4%) and the Control group (27% ± 4%) (*p* < 0.001). There was no significant difference in the proportion of SCs in the proliferative state between the type IV Col group and Control group (*p* = 0.1378).

### 3.2 Assessing the effects of LN, FN, and type IV Col on myelination *in vitro* using SC-DRG coculture

#### 3.2.1 Immunofluorescence staining

**FIGURE 4:**
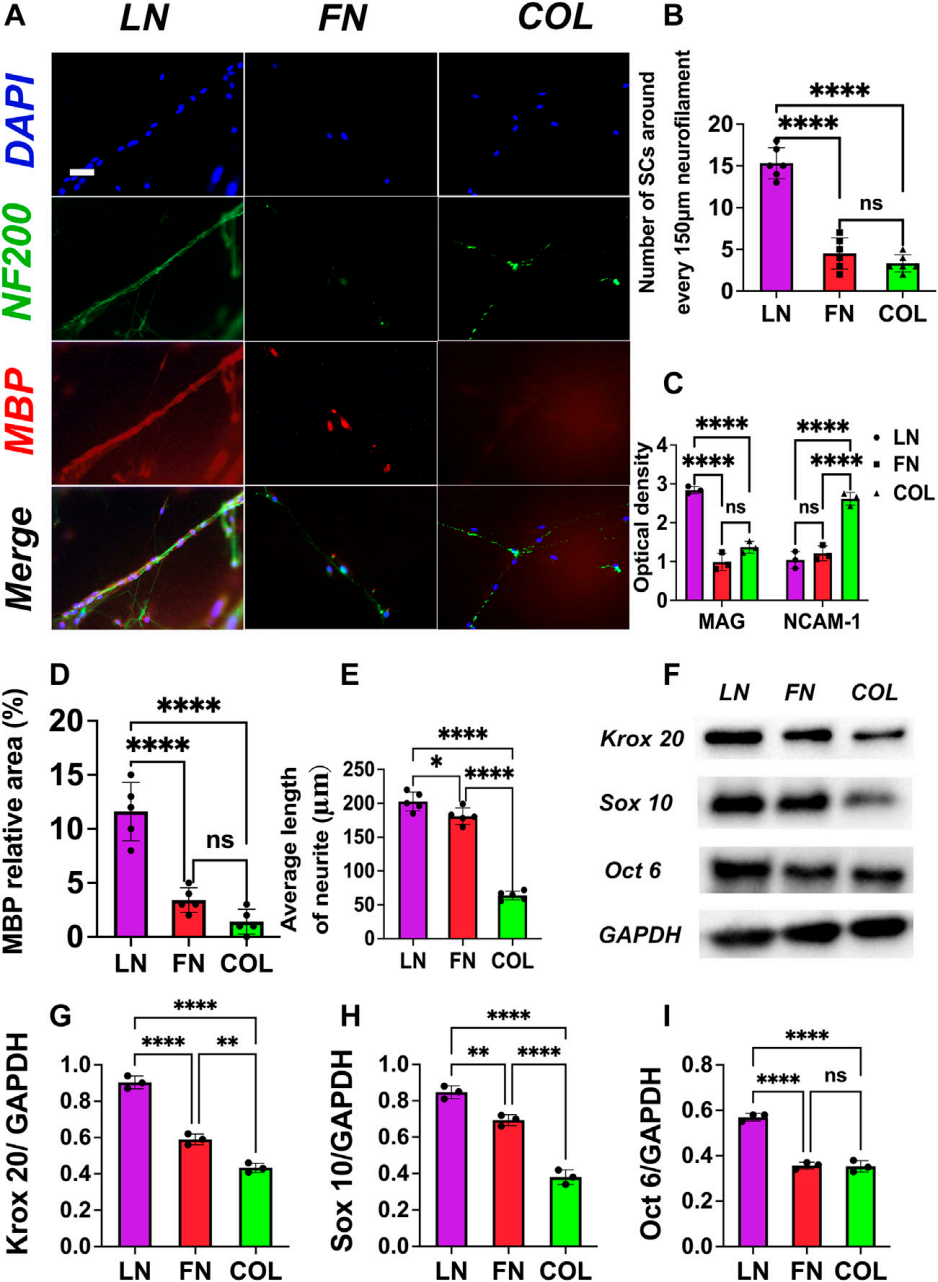
Immunofluorescence staining of SC DRG cocultures: NF-200 (green) and MBP (red) were used to identify neurofilaments and myelin sheaths, respectively. The neurofilaments in the LN group were the thickest, and a large number of SCs were arranged around the neurofilaments **(A)**. Quantitative analysis showed that the number of SCs around neurofilaments per 150 μm length in the LN group was significantly more than that in the FN group and IV Col group (*p* < 0.0001) [**(B)**, *n* = 6]. In the supernatant, the absorbance of MAG (Y-axis) significantly increased and NCAM-1 significantly decreased (*p* < 0.05) [**(C)**, *n* = 3], suggesting a key role of LN in promoting myelin formation. In the FN group, both MAG and NCAM-1 levels were low, a finding that may be related to the local aggregation of FN, thus inhibiting myelination. The average relative area [**(D)**, *n* = 5] of MBP-positive regions (colored red) and the average extension length [**(E)**, *n* = 5] of DRG neuronal processes were measured. The Western blot analysis results **(F)** revealed the presence of transcription factors associated with myelin sheath formation. The expression of myelin-related transcription factors (*n* = 3): Krox 20 **(G)**, Sox 10 **(H)** and Oct 6 **(I)**. *p < 0.05, **p < 0.01, ****p < 0.0001, ^ns^ *p* > 0.05. One-way ANOVA with Tukey’s *post-hoc* test. Scale bar = 20 μm.

Seven days after the SC-DRG coculture system was constructed, immunofluorescence staining was performed on the LN group, FN group, and type IV Col group (Figure 4A): NF200 was stained green (neurofilament), MBP was stained red (myelin sheath), and nuclei were stained blue (DAPI). The number of SCs distributed around neurofilaments per 150 μm length was counted using IPP software, and quantitative analysis was performed using GraphPad Prism 9.0 (Figure 4B). The number of SCs distributed around neurofilaments per 150 μm length in the LN group (15.3 ± 1.9) was significantly more than that in the FN group (4.5 ± 1.9) and type IV Col group (3.3 ± 1.0) (*p* < 0.0001); there was no significant difference between the FN group and type IV Col group (*p* = 0.4518).

#### 3.2.2 Determination of myelin-related proteins in the SC-DRG coculture system and assessment of SC differentiation status by ELISA

Seven days after the construction of the SC-DRG coculture system, MAG (Myelin-associated protein, marker of myelination) and NCAM-1 (Neural cell adhesion molecule 1, immature Schwann cell marker) levels in the supernatant of SC-DRG coculture systems with the LN group, FN group and type IV Col group were measured by ELISA, and the absorbance at 450 nm (OD value, proportional to the concentration of MAG and NCAM-1) was used as the measurement index (Figure 4C). The OD value for the MAG index in the LN group was (2.8 ± 0.1) significantly higher than that in the FN group (1.0 ± 0.2) and type IV Col group (1.4 ± 0.2) (*p* < 0.0001), the OD value for the MAG index in the type IV Col group was slightly higher than that in the FN group (*p* = 0.0645); the OD value for the NCAM-1 index in the type IV Col group (2.7 ± 0.2) was significantly higher than that in the LN group (1.0 ± 0.2) and the FN group (1.2 ± 0.2) (*p* < 0.0001); and the OD value for the MAG index of the FN group was slightly higher than that of the LN group (*p* = 0.5616).

#### 3.2.3 Expression of MBP in co-culture system *in vitro*

Five randomly selected immunofluorescence-stained images were quantitatively analyzed for the relative area of MBP-positive regions (Figure 4A red) in the co-culture system of SCs-DRG with LN, FN, and type IV Col. The results (Figure 4D) showed that the MBP protein expression in the co-culture system of the LN group (11.6% ± 2.7%) was significantly higher than that in the FN group (3.4% ± 1.1%) and the type IV Col group (1.4% ± 1.4%) (*p* < 0.0001). The expression levels in the FN and type IV Col groups were similar (*p* = 0.2307).

#### 3.2.4 Neurite elongation in the SCs-DRG co-culture system of LN, FN and type IV Col groups

25 random DRG neurites were selected in each group (Figure 4A, green), and the average length was quantified (Figure 4E). The results showed that the length of DRG neurite extension in the LN group (202.8 ± 13.9 μm) was significantly longer than that in the FN group (181.0 ± 12.2 μm) (*p* = 0.0251) and type IV Col group (63.8 ± 6.4 μm) (*p* < 0.0001). The length of DRG neurite extension in the FN group was also significantly longer than that in the type IV Col group (*p* < 0.0001).

#### 3.2.5 Expression levels of myelin-related transcription factors (Krox 20, Sox 10 and Oct 6) were evaluated in the co-culture system

The expression of Krox 20 (Figure 4G) in the LN group (0.90 ± 0.03) was significantly higher than that in the FN (0.59 ± 0.02) and type IV Col (0.43 ± 0.02) groups. The expression levels of Sox 10 (Figure 4H) in the LN (0.85 ± 0.03) and FN (0.69 ± 0.02) groups were both were significantly higher than that in the type IV Col group (0.38 ± 0.03). In the LN group, the expression levels of Oct 6 (0.57 ± 0.01) were significantly higher compared to the FN (0.36 ± 0.01) and type IV Col (0.35 ± 0.02) groups (Figure 4I).

### 3.3 SC transwell migration assay

**FIGURE 5:**
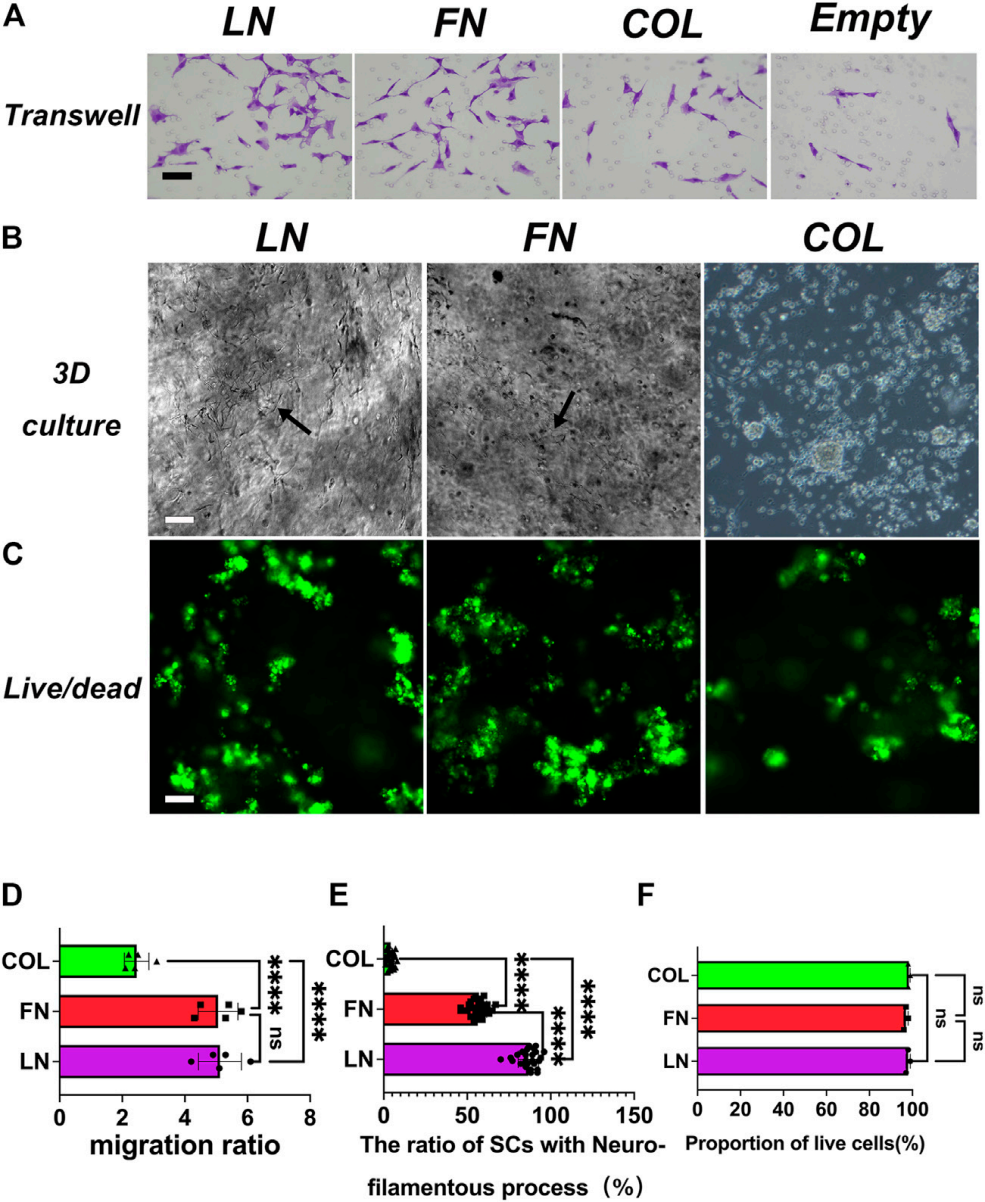
Transwell migration assay of SCs and SCs morphology under 3D culture conditions. The results of the transwell experiment showed that the numbers of SCs that migrated in the LN, FN, and IV Col groups were significantly greater than that in the Control group **(A)**. Growth conditions of SCs under 3D culture conditions were observed using an optical microscope. Cells in the LN and FN groups showed good cell morphology, with elongated processes (black arrows), and cells in the type IV Col group showed mass-like aggregation **(B)**. Live/dead staining analysis of live cells (green)/dead cells (red) in each group **(C)**. Quantitative analysis of cell migration (number of cells in the Control group was taken as reference value 1) [**(D)**, *n* = 5]: the LN group and FN group had similar relative cell migration numbers (*p* > 0.05), and both were significantly more than that in the type IV Col group (*p* < 0.0001). The proportion of SCs with filamentous processes among the total number of cells was assed under 3D culture conditions [**(E)**, *n* = 25]. The proportion of SCs with filamentous processes in the LN group was significantly higher than that in the FN group and type IV Col groups (*p* < 0.0001). Cell survival was assessed [**(F)**, *n* = 3]. The proportion of viable cells in the LN group, FN group and type IV Col group was similar (*p* > 0.05). ****p < 0.0001, ^ns^ *p* > 0.05. Scale bar = 100 µm.

After seeding of SCs in the transwell culture system, the culture was continued for 24 h. The migrated cells in the LN, FN, type IV Col, and Control groups were fixed with 70% (v/v) ethanol and then stained with 0.2% (w/v) crystal violet (Figure 5A). In the Control group, the number of cells that migrated was used as the relative reference value of 1, and then, the relative cell migration numbers in the LN, FN, and type IV Col groups were quantitatively analyzed (Figure 5D): The LN group (5.1 ± 0.7) and FN group (5.0 ± 0.6) had similar relative numbers of migrating cells (*p* = 0.9856), both significantly higher than that in the type IV Col group (2.5 ± 0.4) (*p* < 0.0001).

### 3.4 Evaluation of the effects of LN, FN, and type IV Col on the morphology and cell viability of SCs in a 3-dimensional culture environment

#### 3.4.1 Morphology of SCs

First, sodium alginate hydrogels were developed based on hydrogel composites of LN, FN and type IV Col, and SCs were seeding in the hydrogel so that the cells were evenly distributed and arranged in the 3-dimensional culture environment of the hydrogel. After culturing for 72 h, cell morphology was observed and recorded with an optical microscope (Figure 5B). Most of the SCs in the LN and FN groups exhibited filamentous protrusions (black arrows), while the SCs in the type IV Col group aggregated in clusters. We randomly selected 25 different optical fields of view and quantitatively analyzed the proportion of SCs in the LN, FN, and type IV Col groups with filamentous protrusions (Figure 5E). The proportion of SCs with filamentous protrusions in the LN group was 87% ± 6% on average, significantly higher than that in the FN group (56% ± 5%) and type IV Col group (4% ± 2%) (*p* < 0.0001). The proportion of SCs with filamentous protrusions in the LN group was also significantly higher than that in the type IV Col group (*p* < 0.0001). In Figure 5B (3D culture), the distribution of SCs in the LN and FN groups in the hydrogel is relatively even, with many cells showing spindle-shaped protrusions (indicated by black arrows). This morphology may make it easier for SCs’ cell membranes to contact and wrap around axons to form myelin sheaths. Seventy-two hours after seeding SCs, the cells in the LN and FN groups exhibited neurofilament-like filamentous processes, and the distribution of cells was relatively scattered; in comparison, the cells in the IV Col group were aggregated and distributed in clusters, and the number of SCs with neurofilament-like filamentous processes was very small. However, the quantitative analysis of the proportion of SCs with filamentous protrusions (non-simple circular or quasi-circular in the current field of view) was based on a 2-dimensional field of view under an optical microscope, i.e., there may be some filamentous protrusions of SCs emanating below the plane of the visual field. In short, this phenomenon may be related to the alignment of SCs to form bands of Büngner, the induction of axonal extension, and myelination in later stages.

#### 3.4.2 Evaluation of SCs viability in hydrogel

After culturing SCs in hydrogel systems with composite hydrogels of LN, FN or type IV Col for 72 h, the system was subjected to live (green)/dead (red) staining and recorded using a fluorescence microscope (Figure 5C), and the proportion of viable cells (green) in the composite LN, FN or type IV Col hydrogel system was quantified (Figure 5F). The proportions of viable cells in the LN group (98% ± 1%), FN group (97% ± 1%) and type IV Col group (98% ± 0.6%) were similar (*p* > 0.05).

The proportions of surviving cells in the 3 groups were close to 100%, indicating that LN, FN, and type IV Col can support the survival of cells in sodium alginate hydrogels (3-dimensional space).

### 3.5 Assessment of the effects of LN, FN, and type IV Col on remyelination and nerve function recovery *in vivo*

#### 3.5.1 TEM imaging to assess remyelination

**FIGURE 6:**
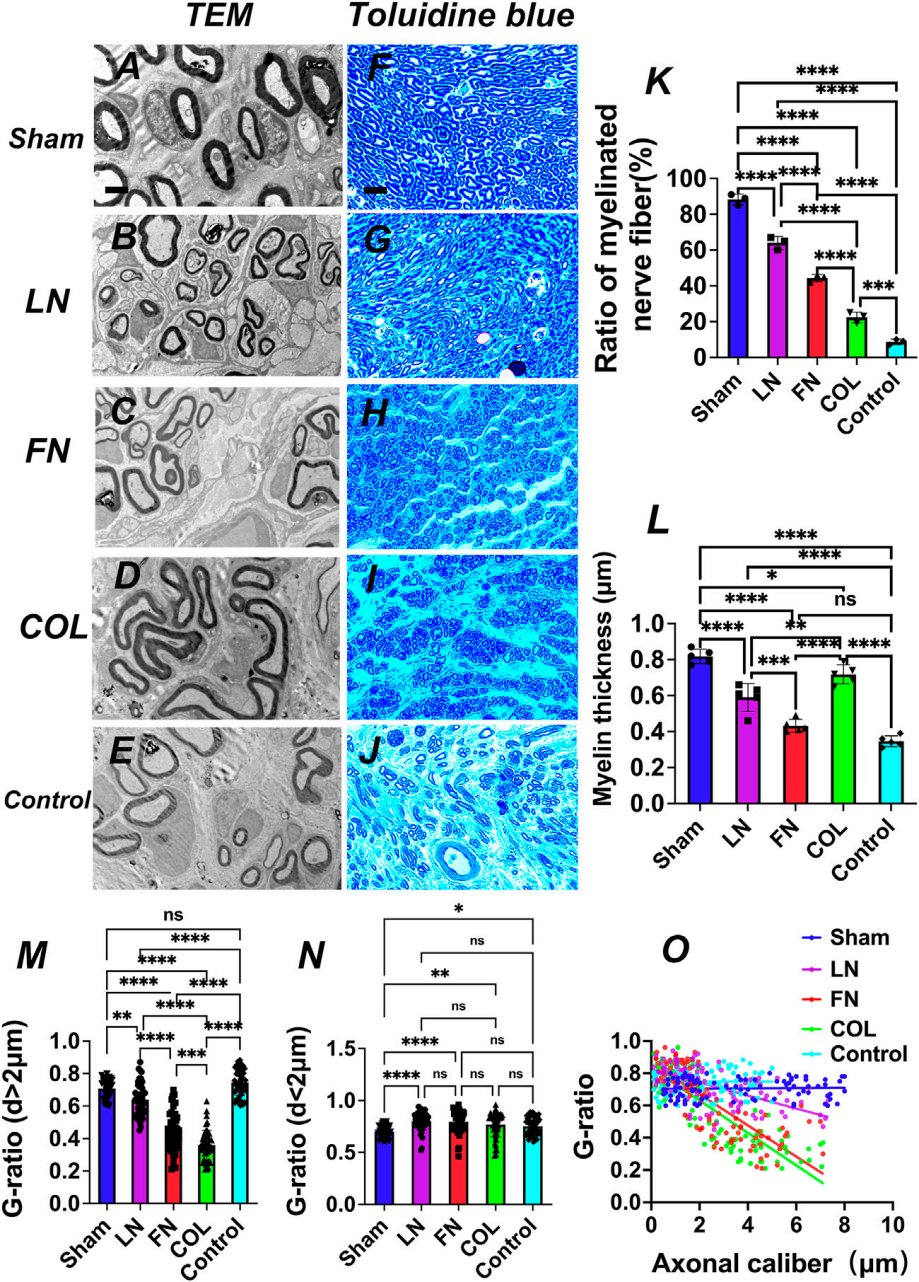
Axon and myelin regeneration at the fourth week after model construction. TEM images **(A–E)** and TB staining images **(F–J)** of axon regeneration at the implanted graft. Quantitative analysis of the proportion of myelinated nerve fibers [**(K)**, *n* = 3]. Quantitative analysis of myelin sheath thickness [**(L)**, *n* = 5]. G-ratio index and nerve function recovery: Quantitative analysis of the G-ratio of axons with a diameter > 2 μm [**(M)**, *n* = 50] and axons with a diameter of < 2 μm [**(N)**, *n* = 50) and a scatter plot based on axon diameter (X-axis) and G-ratio (Y-axis) [**(O)**, *n* = 100]: the G-ratio of the LN group is closer to the best G-ratio (0.6). *p < 0.05, **p < 0.01, ***p < 0.001, ****p < 0.0001, ^ns^ *p* > 0.05. One-way ANOVA with Tukey’s *post-hoc* test. Scale bar **(A–E)** = 2μm, Scale bar **(F–J)** = 700 μm.

After constructing the surgical models for the Sham group (sham-operation group), LN group, FN group, type IV Col group and Control group (implantation of sodium alginate hydrogel without ECM components), sciatic nerve specimens were obtained at the fourth week as was described above (materials and methods), and nerve repair in the sciatic nerve area filled with hydrogel was observed and recorded using TEM (Figures 6A–E) and TB staining (Figures 6F–J).

##### 3.5.1.1 Proportion of myelinated nerves

The quantitative analysis of the proportion of myelinated nerves observed under TEM (Figure 6K) indicated that the average proportions, from high to low, of myelinated nerves in the Sham, LN, FN, type IV Col, and Control groups were 88% ± 3%, 64% ± 4%, 44% ± 2%, 22% ± 3%, and 9% ± 2%, respectively, and the difference was significant (*p* < 0.001). The proportion of myelinated nerves in the LN group was significantly higher than that in the FN group and type IV Col group (*p* < 0.0001). These findings again verified that LN has a stronger effect on promoting remyelination than do FN and type IV Col.

##### 3.5.1.2 Myelin thickness

Myelin sheath thickness was quantitatively analyzed under TEM (Figure 6L). The average thickness of the myelin sheath in the sham group was 0.82 ± 0.04 μm, significantly higher than that in the LN group (0.59 ± 0.08 μm), FN group (0.43 ± 0.04 μm) (*p* < 0.0001), type IV Col group (0.72 ± 0.05 μm) (*p* = 0.0351) and Control group (0.35 ± 0.03 μm) (*p* < 0.0001). The mean myelin thickness in the type IV Col group was significantly higher than that in the LN group and FN group (*p* = 0.005); the mean myelin thickness in the LN group and type IV Col group was significantly higher than that in the Control group (*p* < 0.0001); and the mean myelin thickness in the FN group was slightly higher than that in the Control group (*p* > 0.05).

##### 3.5.1.3 G-ratio of regenerated nerves in the LN, FN, and type IV Col groups

For regenerated axons with axon diameters > 2 μm (Figure 6M), the G-ratio of regenerated nerves in the LN group was 0.64 ± 0.10, significantly higher than that in the FN group (0.44 ± 0.12) and type IV Col group (0.36 ± 0.09) (*p* < 0.001); the G-ratio of regenerated nerves in the FN group was significantly higher than that in the type IV Col group (*p* = 0.0004). For regenerated axons with axon diameters < 2 μm (Figure 6N), there was no significant difference in the G-ratios among the 3 groups (*p* > 0.05) [LN (0.80 ± 0.09), FN (0.79 ± 0.11) and type IV Col (0.77 ± 0.12)]. This indicates that when regenerated axons are thick (axon diameter >2 μm), LN can promote SCs to form myelin sheaths with a more suitable thickness, i.e., closer to the optimal G-ratio value (0.6) (Rushton, 1951). The scatter plots and regression curves for the LN group (red), FN group (blue), and type IV Col group (green) based on axon diameter (X-axis) and corresponding G-ratio (Y-axis) (Figure 6O) show that the G-ratio of the LN group (red) tended to be closer to the optimal G-ratio value.

### 3.6 Immunofluorescence staining to assess nerve and myelin sheath regeneration

**FIGURE 7:**
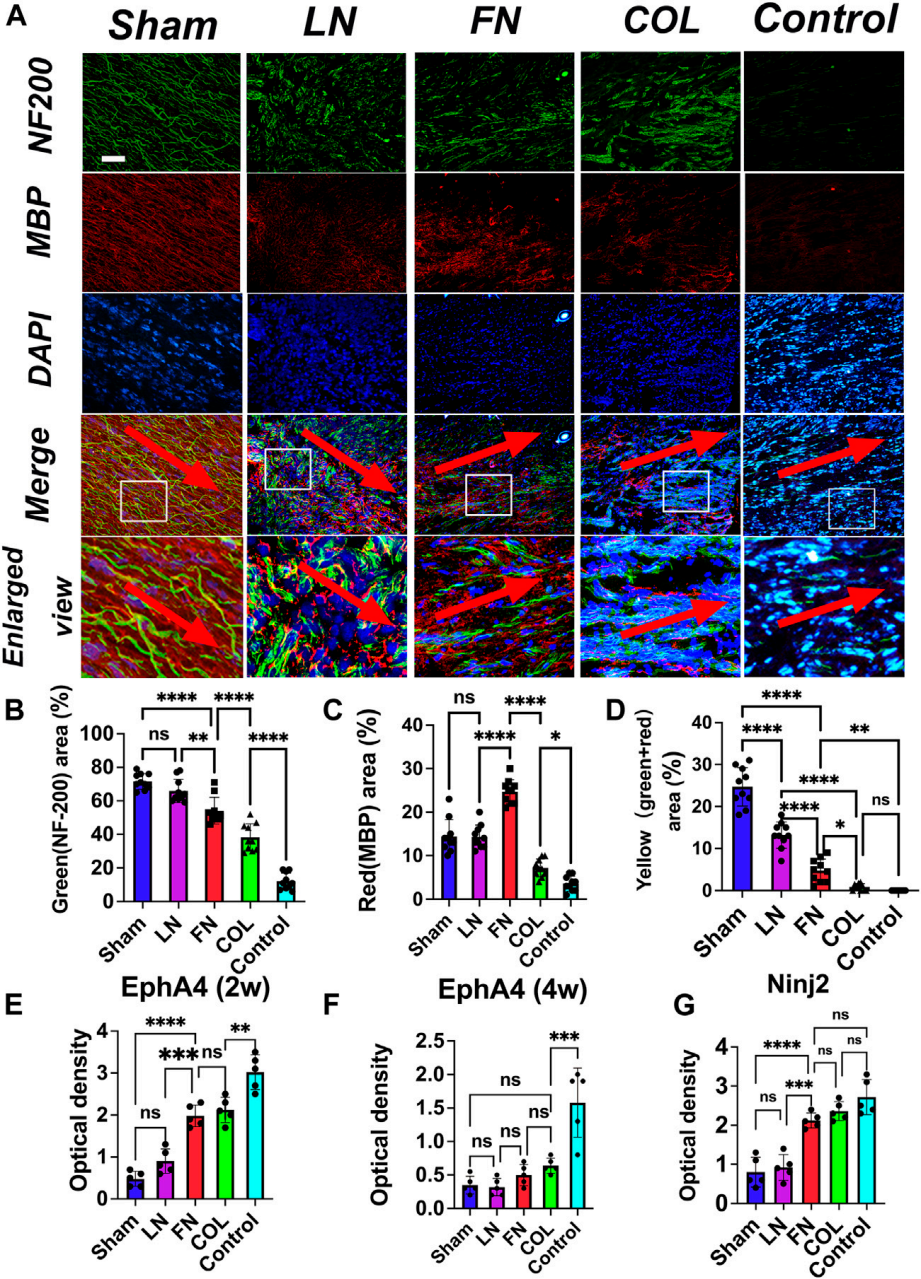
Immunofluorescence assessment of nerve regeneration and remyelination at 4 weeks after model construction. Neurofilaments (NF-200, green) in the LN group were thicker than those in the FN group and type IV Col group, and myelin sheaths were mostly distributed along the neurofilaments (yellow). The neurofilaments in the FN group were significantly thinner than those in the LN group, and although the number of myelin sheaths was greater in the FN group than in the LN group, they were mostly scattered. The numbers of neurofilaments and myelin sheaths in the type IV Col group were significantly less than those in the LN and FN groups **(A)**. The red arrow points from the proximal end of the nerve stump to the distal end. The relative areas of green [neurofilament, **(B)**], red [myelin sheath, **(C)**] and yellow [myelin sheath along the neurofilament, **(D)**] (*n* = 10) in the image were quantified. The level of EphA4 in regenerating nerves was measured at 2 weeks post-surgery **(E)** and at 4 weeks post-surgery **(F)**. Additionally, the level of Ninj2 was measured in regenerating nerves at 4 weeks post-surgery **(G)** (*n* = 5). **p < 0.01, ***p < 0.001, ****p < 0.0001. One-way ANOVA with Tukey’s *post-hoc* test. Scale bar = 100 μm.

Four weeks after surgery, the sections of injured sciatic nerve repaired with the composite LN, FN, and type IV Col hydrogels were stained for immunofluorescence analysis (Figure 7A): neurofilaments were green (NF-200), myelin sheaths were red (MBP), the nucleus was blue (DAPI), and the overlap of neurofilaments (green) and myelin sheaths (red) was yellow (representing myelin sheaths distributed along the nerve). The neurofilaments in the LN group were significantly more hairchested than those in the FN group and type IV Col group, and more myelin sheaths were distributed along the nerve filaments; the thickness of the nerve filaments in the FN group was between that of nerve filaments in the LN group and the type IV Col group, and the number of myelin sheaths along the nerve filaments was significantly lower in the FN group than in the LN group. The neurofilaments in the IV Col group were extremely sparse and slender, and the myelin sheath was significantly reduced.

Quantitative analysis of the proportion (relative area) of the green (neurofilaments), red (myelin sheaths) and yellow (myelin sheath along neurofilaments) regions of the immunofluorescence images revealed that the relative area of the green region (Figure 7B) in the LN group (65.9% ± 7.0%) was significantly larger than that in the FN group (54.1% ± 8.0%) (*p* = 0.0056) and type IV Col group (38.2% ± 8.2%) (*p* < 0.0001) and that the relative area of green in the FN group was also significantly larger than that in the type IV Col group (*p* = 0.0003). The relative area of neural fibers in all three groups was found to be smaller than that of the Sham group (71.6% ± 4.7%), and significantly larger than that of the COL group (12.1% ± 5.0%). The relative area of red (Figure 7C) in the FN group (24.7% ± 2.8%) was significantly larger than that in the LN group (14.3% ± 2.8%) and IV Col group (7.2% ± 2.0%) (*p* < 0.0001), and the relative area of red in the LN group was also significantly larger than that in the type IV Col group (*p* < 0.0001) and Control group (3.6% ± 1.7%). The relative area of the red region was similar between the LN group and Sham group (14.4% ± 3.9%). The relative area of yellow (Figure 7D) in the LN group (13.2% ± 3.1%) was significantly larger than that in the FN group (4.8% ± 2.7%) and type IV Col group (0.9% ± 0.8%) (*p* < 0.0001), and the relative area of yellow in the FN group was also significantly larger than that in the type IV Col group (*p* = 0.004). The Control group did not observe any myelin sheath regeneration along the nerve fibers. Furthemore, the Sham group exhibited a significantly larger relative area of yellow staining (24.7% ± 4.6%) compared to the other four groups.

### 3.7 The content of EphA4 in sciatic nerve graft specimens was determined by ELISA

Two weeks and 4 weeks after surgery, the sciatic nerve graft specimens were taken from rats, and the EphA4 content in the regenerating nerves was measured. Two weeks after surgery (Figure 7E), the EphA4 content in the LN group (0.90 ± 0.29) was significantly lower than that in the COL group (2.12 ± 0.30) (*p* < 0.0001) and the FN group (1.98 ± 0.26) (*p* = 0.0002); Four weeks after surgery (Figure 7F), the EphA4 content in the LN group (0.32 ± 0.13) was significantly lower than that in the COL group (0.64 ± 0.11) (*p* = 0.0074), and slightly lower than that in the FN group (0.50 ± 0.16) (*p* > 0.05). In the Sham group, the expression level of EphA4 remained consistently low (2 weeks: 0.48 ± 0.18; 4 weeks: 0.35 ± 0.13). In contrast, the expression of EphA4 in the Control group (2 weeks: 3.02 ± 0.41; 4 weeks: 1.58 ± 0.12) remained consistently higher than in the other groups.

### 3.8 The content of Ninj2 in sciatic nerve graft specimens was determined by ELISA

Four weeks after the surgery, the sciatic nerve grafts were harvested from rats and the levels of Ninj2 in the regenerated nerves were measured. The results showed that, at 4 weeks post-surgery (Figure 7G), the Ninj2 levels in the LN group (0.92 ± 0.33) were significantly lower than those in the COL group (2.36 ± 0.24), the FN group (2.12 ± 0.19) (*p* < 0.0001) and Control group (2.72 ± 0.45). The Ninj2 levels in the COL and FN groups were similar (*p* = 0.3422). And The Ninj2 levels in the Sham (0.80 ± 0.38) and LN groups were similar (*p* > 0.05).

### 3.9 Assess the atrophy of the muscles innervated by the sciatic nerve

**FIGURE 8:**
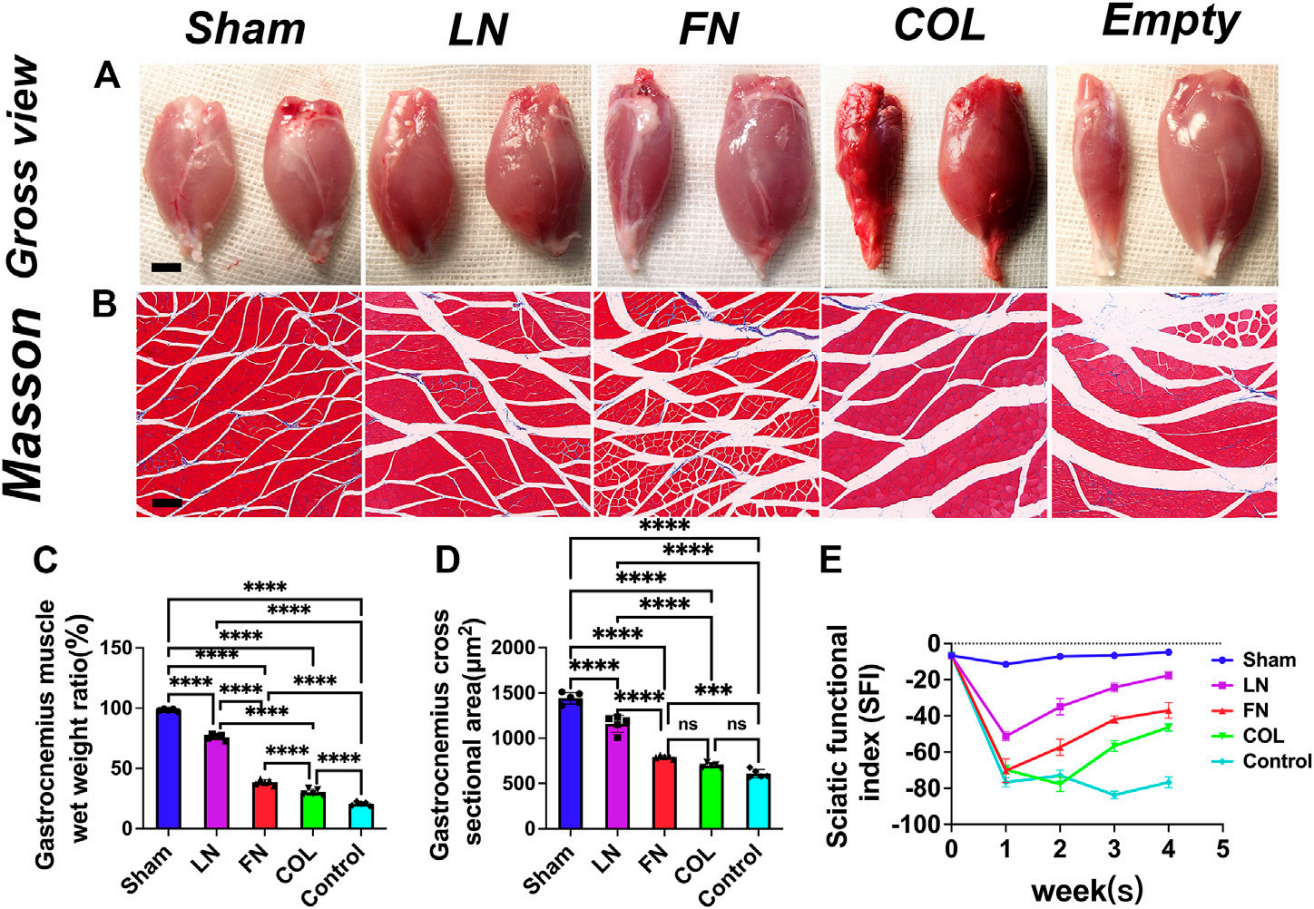
Atrophy of innervated muscles at 4 weeks after model construction. Representative gross view of gastrocnemius muscle in each group **(A)**: the injured side is on the left, and the normal side is on the right. Masson staining of the gastrocnemius muscle on the injured side of the nerve **(B)**. Wet weight ratio of gastrocnemius muscle (injured side/normal side) [**(C)**, *n* = 5]. Average cross-sectional area of muscle fibers in each group [**(D)**, *n* = 5]. Sciatic functional index (SFI) was measured in each group after surgery [**(E)**, *n* = 3]. Scale bar **(A)** = 5 mm. Scale bar **(B)** = 50 μm ***p < 0.001, ****p < 0.0001, ^ns^ *p* > 0.05. One-way ANOVA with Tukey’s *post-hoc* test.

Four weeks after the operation, the gastrocnemius muscle on the left and right sides of the rat was removed (Figure 8A). There was no significant difference in the appearance of the gastrocnemius muscle on the experimental side between the LN group and the sham group. The gastrocnemius muscle on the experimental side in rats in the FN group, type IV Col group and Control group all had different degrees of atrophy. After removing the muscles, they were quickly weighed [expressed as the weight of the muscle on the experimental side/the weight of the muscle on the normal side (%)], and GraphPad Prism 9 software was used to conduct a quantitative analysis (Figure 8C). The wet weight ratio of the gastrocnemius muscle in the sham group (98.4% ± 0.9%) was significantly greater than that in the LN group (76.0% ± 2.3%), FN group (38.4% ± 2.2%), type IV Col group (30.2% ± 2.6%) and Control group (20.5% ± 1.6%) (*p* < 0.0001). The wet weight ratio of gastrocnemius muscle in the LN group, FN group, and type IV Col group was significantly greater than that in the Control group (*p* < 0.0001), and the wet weight ratio of the gastrocnemius muscle in the LN group was also significantly greater than that in the FN group and the IV Col group (*p* < 0.0001).

After obtaining weights, Masson staining was performed (Figure 8B). The average cross-sectional area of the gastrocnemius muscle on the experimental side in rats (Figure 8D) in the FN group (792.6 ± 18.3 μm^2^), type IV Col group (697.2 ± 25.4 μm^2^) and Control group (608.6 ± 45.5 μm^2^) was significantly smaller than that in rats in the LN group (1159.0 ± 91.7 μm^2^) and the sham group (1440.6 ± 64.4 μm^2^) (*p* < 0.0001); in addition, the LN group was significantly smaller than the sham group (*p* < 0.0001). The average cross-sectional area of the gastrocnemius muscle on the experimental side in rats in the LN group (*p* < 0.0001) and FN group (*p* = 0.0004) was significantly larger than that in rats in the Control group, and that in rats in the type IV Col group was slightly larger than that in rats in the Control group (*p* = 0.1286).

### 3.10 Dynamically monitoring the sciatic functional index (SFI) after surgery

The SFI in the Sham group remained close to 0 (normal function) throughout the 4-week postoperative period, while the SFI in the Control group remained close to −100 (complete loss of function). In the first week, all groups except for the Sham group showed a significant decrease in SFI. In the LN, FN, and COL groups, the SFI gradually improved in weeks 2–4, with LN showing the fastest rate of recovery followed by FN and then COL (Figure 8E).

## 4 Discussion

The ECM provides adhesion sites and structural support for SC migration (Gao et al., 2013; Leclech et al., 2020) and is an indispensable element for cell migration. Basal lamina proteins, as components of the ECM, for example, type IV collagen, fibronectin and laminin, are known to influence cell adhesion (Gao et al., 2013). SCs express integrin heterodimers that act as receptors for laminin and fibronectin, which are present in the basal lamina. LN can directly interact with its receptors on SCs, facilitating cell adhesion (Durbeej, 2010). For example, Integrin α6β4, a laminin receptor synthesized by SCs, is critical for cell migration as it is displayed opposite to the basal lamina (Su et al., 2021).

ECM surrounding undamaged SCs can facilitate their transition and migration towards the injured site for early nerve repair, and act as a physical guide for nerve regeneration. Moreover, it can also secrete neurotrophic factors for regenerating axons. However, when establishing 3D cultures of SCs with ECM, some differences were observed. LN and FN groups displayed elongated cell processes 3 days after implantation, while cells in type IV Col group showed shorter extension distance and aggregation compared to LN and FN groups. Increasing the concentration of type IV Col in the hydrogel and extending the observation time did not alter the results (data not shown). Collagen had a weaker effect than the other two non-collagen glycoproteins in promoting the adhesion process of SCs in 3D space.

Following lesion-induced Wallerian degeneration, SCs start to proliferate, forming longitudinal cell strands termed bands of Büngner (Stoll and Müller, 1999; Ribeiro-Resende et al., 2009; Panzer et al., 2020; Ramesh et al., 2021). Studies have shown that the ECM can not only mediate cell adhesion but also guide cell extension. LN induces alignment-oriented extensions and spindle-shaped morphology of SCs, which may facilitate the formation of Büngner bands (Figures 2B, 4A) (Ribeiro-Resende et al., 2009) (the linear bands of longitudinally aligned SCs) to better induce axonal extension. Type IV Col is a member of the reticular collagen group and is a common component of the basement membrane (Chen et al., 2015). Biomaterials are designed to induce longitudinally aligned Schwann cells (SCs) and form bands of Büngner, facilitating axonal regrowth. The ECM plays a crucial role in the formation of bands of Büngner, although the mechanism remains unclear. Aligned SCs provide indispensable pathways for guided axonal regrowth, and their migration ability enhances the formation of bands of Büngner, which induce neural axon extension. Transwell experiments showed that LN and FN significantly promote SC migration, with LN having a more significant effect. This may facilitate SC movement to appropriate locations and improve the formation of bands of Büngner.

After peripheral nerve injury, SCs undergo transformation processes including dedifferentiation, proliferation, and redifferentiation into promyelinating states. The myelin SCs dedifferentiate into a new state of SCs known as LiSCs, which possess immature SC features and activate repair-related phenotypes, forming repair SCs (Jessen and Mirsky, 2016). Such SCs are able to proliferate and form bands of Büngner. When in contact with thick-caliber axons, LiSCs differentiate into myelinating SCs (a promyelinating phenotype) and segregate the associated axon, a process known as “radial sorting” (Balakrishnan et al., 2020). Studies have shown that the interaction between SCs and the ECM is crucial for remyelination in this process. Evidence suggests that LN is a necessary factor in the process of radial sorting (Court et al., 2006). Schwann cell-specific deletion of gamma1-laminin blocks axonal sorting almost completely (Yu et al., 2005).

In this study, upon SC-DRG coculture, significant upregulation of MAG (myelin-specific proteins) and significant downregulation of NCAM-1 (an immature Schwann cell marker) were observed in the LN group (Figure 4C). We speculate that LN may play a role in promoting the maturation of SCs. Some studies have found that without LN, SCs can migrate along axons, populate peripheral nerves, and proliferate normally but cannot differentiate into a myelinating phenotype (Chen and Strickland, 2003). In addition, SCs encapsulate axons and secrete neurotrophic factors to promote axon regeneration and guide extension. As is known to all, missing type IV Col and the assembly of fibrillar and basement membranes in SC ECM structures fail to impair remyelination and cause axonal damage, thus altering axonal functions. During co-culture, the expression of relevant proteins representing myelin formation was not significantly elevated in the COL group, and no obvious remyelination was observed in *in vivo* experiments. Therefore, we speculate that type IV Col is more likely to play a role in stabilizing myelin after its formation. Unexpectedly, FN group showed the lowest remyelination with significantly reduced expression of immature Schwann cell marker and low level of MAG (Figure 4C). The deposition of FN may interfere with myelin sheath formation, possibly *via* a mechanism similar to that in the central nervous system (Su et al., 2021). MBP is essential for primary myelin formation and regulation, as well as the appropriate thickness and density of myelin in both the CNS and PNS. LN outperforms other ECM proteins in promoting proper myelin regeneration. CNS studies have shown that the axonal surface adhesion molecule L1 stimulates the synthesis of MBP *via* the Src family kinase Fyn, which is present in oligodendrocytes (Wake et al., 2011). L1 induces Fyn activation (White et al., 2008; Laursen et al., 2009), leading to hnRNP-A2 phosphorylation and release of hnRNP-A2 and hnRNP-E1 from mRNA transport granules (White et al., 2008), as well as integrin signaling, which activates Fyn (Wake et al., 2011) and interacts with another mRNA-binding protein hnRNP-K (Laursen et al., 2011), affecting the synthesis of MBP. Our study found a higher number of SCs aligning along axons in the LN group (Figure 4A), indicating that LN may promote myelin regeneration by binding to Schwann cell surface receptors, promoting chemotaxis, and creating an optimal environment for S-A interactions and downstream pathways that regulate MBP expression. Similar molecular pathways may regulate myelin regeneration in both the CNS and PNS, possibly acting alone or in combination to control MBP expression. The results of the Western blot experiment also indicated that LN may play a role by promoting the transcription factors (Krox 20, Sox 10 and Oct 6) that positively regulate myelin regeneration.

The length of DRG neurite extension in the co-culture system of SCs and DRG neurons was significantly longer in the LN group than in the FN group and the type IV Col group (Figure 4E), indicating that LN has a stronger ability to promote neurite elongation compared to the other two groups. One possibility is that LN directly promotes axon extension by binding to surface receptors on neurons, such as the β1 integrin-laminin signaling pathway, which has been shown to directly promote neurite outgrowth of dorsal root ganglion neurons co-cultured with Schwann cells (Bixby et al., 1988). On the other hand, the contact between SCs and neurons is an important condition for axonal extension (Armstrong et al., 2007). The LN group had a large number of SCs arranged along the neurite (Figure 4A), and these SCs arranged along the neurite may directly or indirectly promote the release of neurotrophic factors by SCs, thereby promoting axonal extension.

*In vivo* effects of ECM components on remyelination remain unclear despite significant impacts on SCs’ behavior demonstrated *in vitro*. To bridge this gap, we utilized hydrogel complexes with purified ECM. The S-A interaction is essential for nerve regeneration, and axon regeneration quality is directly linked to myelin sheath regeneration. LN promoted the regeneration of myelin sheath and axon and led to better nerve function recovery compared to other groups (Figures 6, 8), indicating its significant role in promoting regeneration. By monitoring the SFI of rats at multiple time points after surgery, we indirectly evaluated the degree of nerve recovery. The results also showed that LN had a significant advantage in improving the rate of nerve function recovery after peripheral nerve injury (Figure 8E). Immunofluorescence staining results also showed (Figure 7A) that the myelin sheath tended to regenerate, distribute and arrange along neurofilaments and that S-A interactions generated by such distribution may further promote the regeneration of axons. Interestingly, FN did not inhibit *in vivo* remyelination, contrary to its effect *in vitro*. This may be attributed to the high local concentration of FN in 2D conditions or degradation of the hydrogel. *In vivo*, FN facilitated axonal regeneration and remyelination, but the regenerated myelin sheath was not properly aligned with the neurofilament, which may hinder further nerve repair and functional recovery. Moreover, nerve filaments were thinner. Thus, the role of FN in nerve regeneration *in vivo* is limited. Type IV Col group exhibited increased myelin sheath thickness, but not significant improvement in the number of myelinated nerve fibers, suggesting that Type IV Col may stabilize myelin after myelination. Type IV Col may indirectly promote SC myelin formation by enabling the assembly of basal lamina (Eldridge et al., 1989; Court et al., 2006), potentially explaining why myelination and axon regeneration were not ideal in the type IV Col group.

Myelin sheath formation is influenced by both positive and negative regulatory factors, which can be affected by ECM proteins. This can have direct implications for myelin regeneration after PNI. EphA4, a member of the Eph tyrosine kinase receptor family, has been shown to regulate cell-cell interactions through ephrins and may play a role in this process (Chen et al., 2019). *In vivo* experiments demonstrated that EphA4 is upregulated in SCs following nerve crush injury and downregulated during demyelination, indicating its negative regulation of myelination. Our findings suggest that ECM proteins may inhibit EphA4 expression in the peripheral nervous system post-injury, reducing downstream pathway activation that inhibits myelination. LN exhibited the most substantial inhibitory effect on EphA4 expression among the tested ECM proteins, particularly in the early stages of myelination.

Ninjurin 2, a surface adhesion molecule encoded by the Ninjurin 2 gene, has been identified as a negative regulator of the myelination network in the PNS (Sun et al., 2022). Ninj2 interacts with ITGB1 on SC membrane to inhibit the laminin-integrin pathway. We speculate that there may be a mutual inhibition relationship between Ninj2 and LN *in vivo*. LN not only inhibits Ninj2 expression and weakens its inhibitory effect on the laminin-integrin pathway but also directly promotes this pathway, which positively regulates myelin formation.

## 5 Limitation

In investigating the impact of three ECM proteins on myelin regeneration, we selected specific concentrations that maintained optimal cell viability. The complex and intriguing effects of ECM at different concentrations on SCs’ biological behavior and myelin formation were not extensively explored and remain a topic for future investigation.

## 6 Conclusion

In this experiment, we analyzed and compared the effects of LN, FN, and type IV Col on the biological behavior of SCs and their effects on remyelination after PNI and further clarified their unique roles in the process of remyelination. Further research is necessary to explore the underlying mechanisms.

## Data Availability

The raw data supporting the conclusion of this article will be made available by the authors, without undue reservation.

## Supplementary material

SUPPLEMENTARY FIGURE S1 The characterization of S16 cell line. Immunofluorescent staining of S100 **(A)** and p75 **(B)**.

SUPPLEMENTARY FIGURE S2 Degradation curve of sodium alginate hydrogel in vitro.
