# Supplementary figures and images for: Effects of ECM proteins (laminin, fibronectin, and type IV collagen) on the biological behavior of Schwann cells and their roles in the process of remyelination after peripheral nerve injury
Source: Front Bioeng Biotechnol. 2023 Mar 24;11:1133718. doi: 10.3389/fbioe.2023.1133718 (PMC10080002; doi:10.3389/fbioe.2023.1133718)

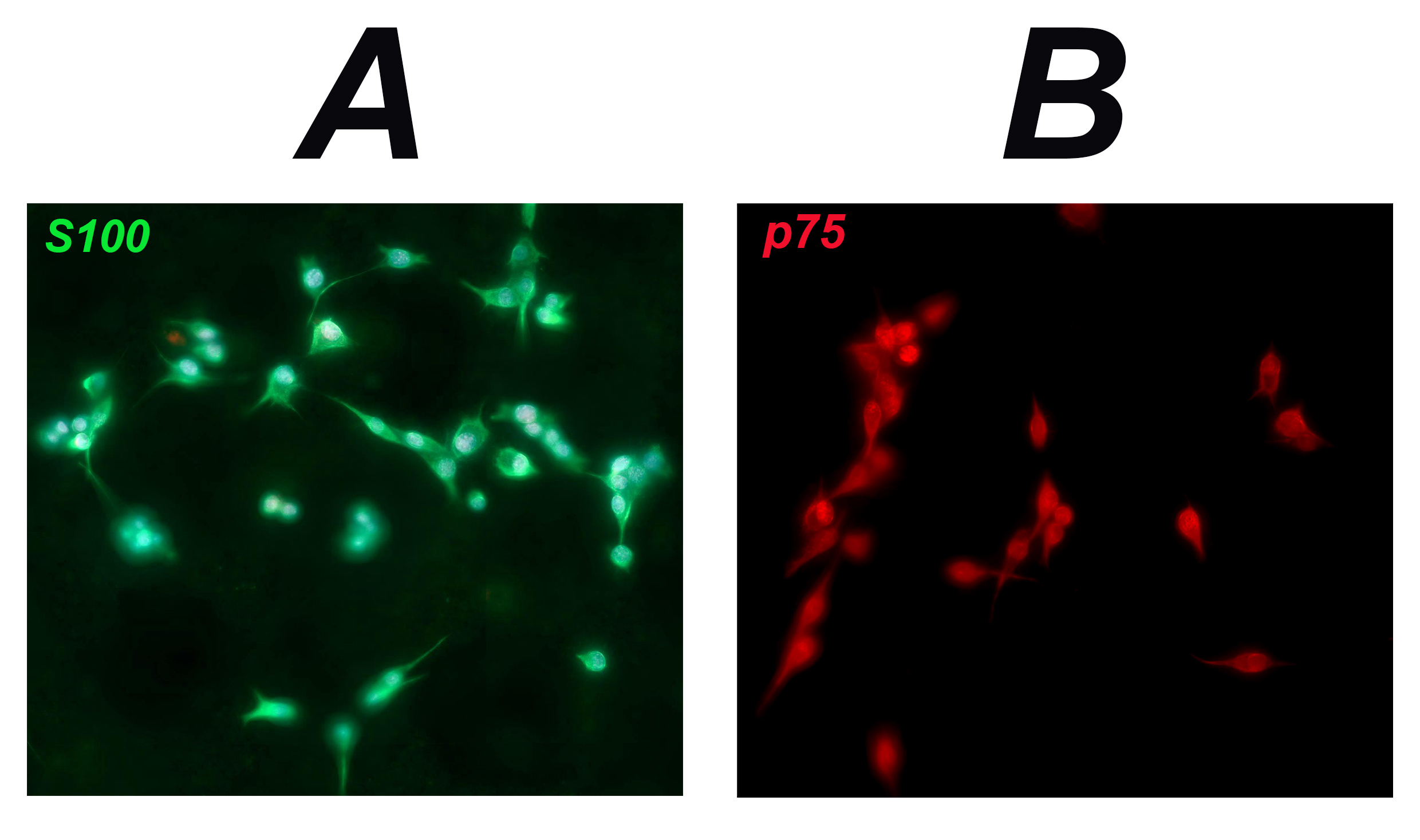

Supplement: Supplementary file 1 [file Image1.JPEG]

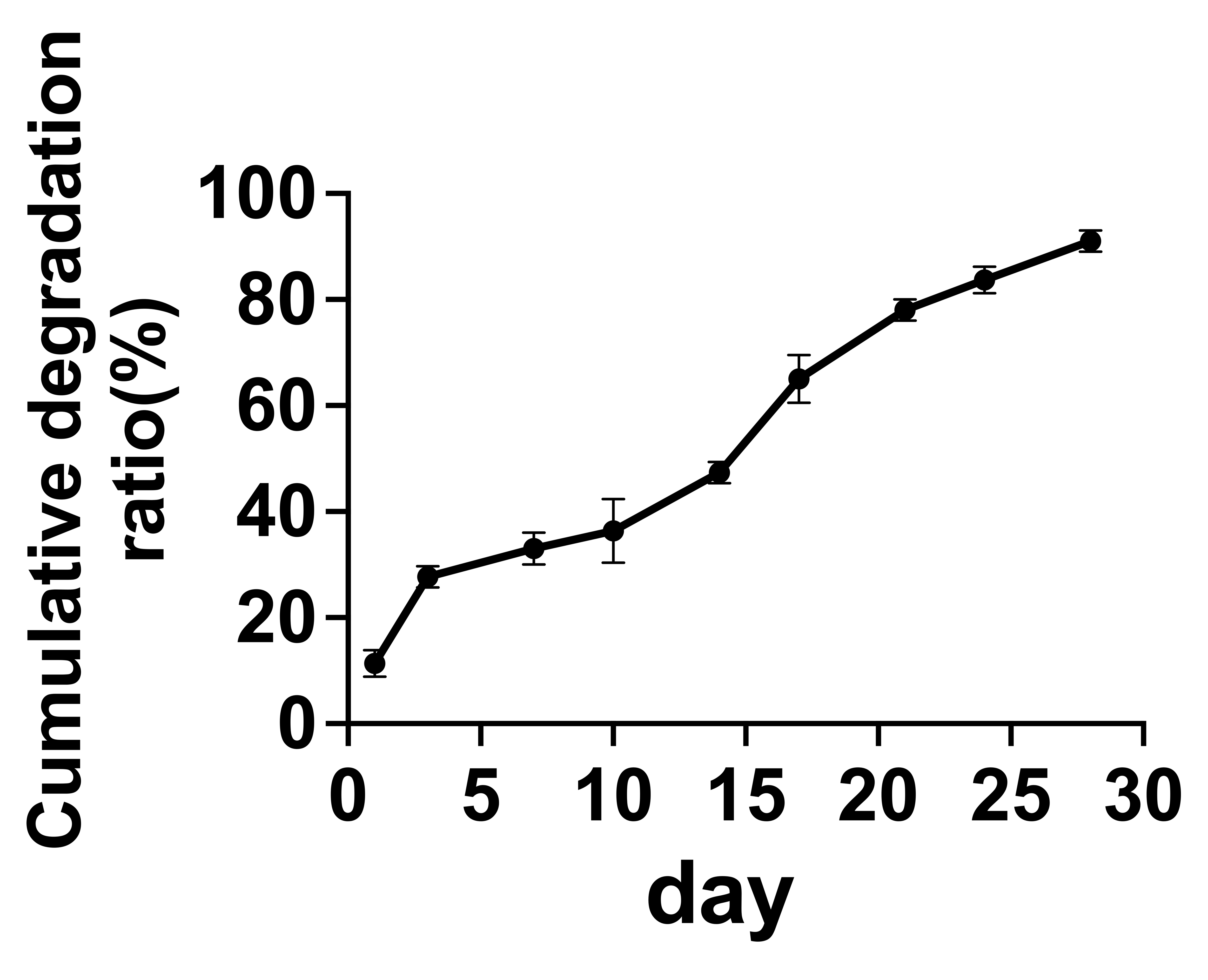

Supplement: Supplementary file 2 [file Image2.JPEG]
